# Topoisomerase 2B Decrease Results in Diastolic Dysfunction *via* p53 and Akt: A Novel Pathway

**DOI:** 10.3389/fcvm.2020.594123

**Published:** 2020-11-06

**Authors:** Rohit Moudgil, Gursharan Samra, Kyung Ae Ko, Hang Thi Vu, Tamlyn N. Thomas, Weijia Luo, Jiang Chang, Anilkumar K. Reddy, Keigi Fujiwara, Jun-ichi Abe

**Affiliations:** ^1^Department of Cardiovascular Medicine, Heart and Vascular Institute, Cleveland Clinic Foundation, Cleveland, OH, United States; ^2^Department of Cardiology, Division of Internal Medicine MD Anderson Cancer Center, Houston, TX, United States; ^3^Texas A&M Health Science Center, Institute of Biosciences and Technology, Houston, TX, United States; ^4^Department of Medicine, Baylor College of Medicine, Houston, TX, United States

**Keywords:** diastolic dysfunction (DD), topoisomerase 2 beta (TOP2b), Akt, p53, echocardiagraphy

## Abstract

Diastolic dysfunction is condition of a stiff ventricle and a function of aging. It causes significant cardiovascular mortality and morbidity, and in fact, three million Americans are currently suffering from this condition. To date, all the pharmacological clinical trials have been negative. The lack of success in attenuating/ameliorating diastolic dysfunction stems from lack of duplication of myriads of clinical manifestation in pre-clinical settings. Here we report, a novel genetically engineered mice which may represents a preclinical model of human diastolic dysfunction to some extent. Topoisomerase 2 beta (Top2b) is an important enzyme in transcriptional activation of some inducible genes through transient double-stranded DNA breakage events around promoter regions. We created a conditional, tissue-specific, inducible Top2b knockout mice in the heart. Serendipitously, echocardiographic parameters and more invasive analysis of left ventricular function with pressure–volume loops show features of diastolic dysfunction. This was also confirmed histologically. At the cellular level, the Top2b knockdown showed morphological changes and molecular signaling akin to human diastolic dysfunction. Reverse phase protein analysis showed activation of p53 and inhibition of, Akt, as the possible mediators of diastolic dysfunction. Finally, activation of p53 and inhibition of Akt were confirmed in myocardial biopsy samples obtained from human diastolic dysfunctional hearts. Thus, we report for the first time, a Top2b downregulated preclinical mice model for diastolic dysfunction which demonstrates that Akt and p53 are the possible mediators of the pathology, hence representing novel and viable targets for future therapeutic interventions in diastolic dysfunction.

## Introduction

Heart Failure remains one of the greatest health crises in the western world. It is estimated that life-time risk of developing heart failure is approximately 25–45% as per recent Heart Disease and Stroke Statistics: update 2019 (1). Of those diagnosed, heart failure with preserved ejection fraction (EF; mostly, diastolic dysfunction) represents more than half of the cohort. Despite this tremendous burden, our knowledge about diastolic dysfunction is quite limited. Over the years we have become proficient at diagnosing the disease, however, we lack the molecular understanding of the disease process and most importantly, the therapeutic intervention that can decrease this burden. Historically, dysfunction in nitric oxide (2), intracellular calcium dysregulation (3), and/or aberration in neuro-hormonal mechanisms (4) are some of the proposed pathways implicated in diastolic dysfunction. More recently, Dr. Hill's group proposed a two-hit model which entails metabolic and mechanical stress as potential precipitators of diastolic dysfunction. They highlighted that a combination of systemic inflammation, inducible nitric oxide synthase activation, nitrosative stress, and suppression of unfolded protein response triggers the precipitation of diastolic dysfunction. However, the proposed model failed to determine the aging aspect of development of diastolic dysfunction. Moreover, various clinical trials conducted to influence the afore-mentioned pathways have been negative (5). The key component in the knowledge gap is due to the non-existence of an animal model which can be directly translatable to the human population.

We published previously that anthracyclines disrupt the catalytic activity of Top2b, resulting DNA double-stranded breaks, initiating a cascade of events, resulting in defective mitochondrial biogenesis and reactive oxygen species generation, thus precipitating cardiomyopathy (6). Additionally, cardiomyocyte-specific deletion of Top2b ameliorated anthracycline-mediated cardiomyopathy. Therefore, we were interested in the role of Top2b in cardiac function. Serendipitously, we identified a possible new genetic model for diastolic dysfunction.

## Methods

### Generation of Cardiomyocyte-Specific Top2b^-/-^ Mice

The use of animals, including all treatments, was approved by the Institutional Animal Care and Use Committees of the University of Texas MD Anderson Cancer Center. The generation of cardiomyocyte specific Tob2b-deleted mice has been described elsewhere. Briefly, the mouse carrying floxed Top2b (loxP site flanked three Top 2b exons) were crossed with α-MHC promoter–driven Cre flanked with a mutated mouse estrogen receptor ligand–binding domain (MerCreMer). This cassette was responsive to tamoxifen and insensitive to estrogen as published elsewhere (6). The final genotype generated was α-MHC-MerCreMer Top2b^flox/flox^ mice. α-MHC-MerCreMer Top2b^+/+^ and α-MHC-MerCreMer Top2b^+/flox^ mice were used as controls. Specific primers provided by the vendor (Jackson Labs) were used to identify the Cre allele. For the identification of the wild-type Top2b (~820 bp) and the floxed Top2b (~600 bp) alleles, primers UpBglII (5′-ATATGGTACAGCAACAAAGCATTTGA-CATA-3′), and PacIR (5′-TCATTGGGAGGCCAGAGCATC-3′) were used in PCR-based genotyping. For our complete analysis, both male and female sex were used in the study. Each group had roughly the same number of each sexes.

### Echocardiography

Mice were anesthetized with 1.5% isoflurane and imaged in the supine position using a Vevo 2100 Imaging System with a 40-MHz linear probe (Visualsonics, Toronto, Canada). Core temperature was maintained at 37°C. Heart rates were kept consistent between experimental groups (400–500 bpm). ECG monitoring was obtained using limb electrodes. A standard 2D echocardiographic study was initially performed in the parasternal long-axis as well as short-axis view for assessment of LV dimensions and systolic function. Image depth, width, and gain settings were used to optimize image quality. Doppler flow profiles were acquired using pulsed wave Doppler in the parasternal long axis. The sample volume was placed close to the tip of the mitral leaflets parallel to the blood flow in order to record maximal transmitral flow velocities. Tissue Doppler imaging in the parasternal view was performed by placing the pulsed wave Doppler at the lateral corner of the mitral annulus. E^/^ at the lateral wall was noted as peak mitral annular velocity during early filling.

The strain was calculated using Vevostrain software (4). Briefly, tracking points were placed by Vevo2100 Imaging Software on the endocardial and epicardial border in the parasternal long axis. The track points were adjusted to follow the contours of the myocardium. Subsequent frame by frame tracking through the cardiac cycle allowed automatic calculation of strain and strain rates. The software divides the LV into six segments and calculates longitudinal and radial strain and strain rates for each segment, as well as, overall mean values. The data presented in this study are the mean global longitudinal and radial strain rate values.

All views were digitally stored in cine loops consisting of 300 frames. Subsequent analyses were performed off-line by experienced sonographers who were blind to the type of mouse model. LV volumes and EF were obtained using the standard 2D quantification software. Inter-observer and intra-observer variability for indices of diastolic function were evaluated from 10 randomly selected echocardiographic studies, analyzed in a blinded manner on two separate occasions by the same observer (intra-observer variability) and by another independent observer (inter-observer variability). Roughly 9–10 animals were used to generate data in each group.

### Pressure–Volume Loop

Hemodynamic parameters were obtained using Millar catheter (PVR-1045; Millar Instruments, Houston, TX, USA) in an open-chest technique. Mice were anesthetized with 1.5% isoflurane by inhalation. The mice were intubated to control for respiratory variation during pressure–volume (PV) analysis. The right internal jugular vein was cannulated for intravenous fluid to compensate for volume loss. Animal temperatures were monitored and maintained at 37°C while PV loops were recorded with Labview 7.1 software (National Instruments, Austin, TX, USA). PV data were then analyzed with Pressure–Volume Analysis Software (PVAN Millar Instruments, Houston, TX, USA). For volume calibration, standard volumes were determined with echocardiography. Left ventricle was accessed with abdominal incision and apical puncture was performed with a 23-gauge needle. Successful cannulation of the left ventricle was done through the incisional port made by the needle. After 30 min of stabilization and intravenous fluid resuscitation, measurements were made. A snare was designed and used for IVC occlusion. Roughly 6–7 animals were used to generate data in each group.

### Trichrome Staining/Analysis

Trichrome staining was performed with Trichrome Stain (Masson) Kit (Abcam, Cambridge, MA USA). The manufacturer's instructions were used. Briefly, the cryosections were thawed and Bouin's solution was used to fix the samples. Weigert's Iron Hematoxylin was used for nuclear staining, Biebrich Scarlet/Acid Fuchsin Solution for cytoplasmic staining and aniline blue for collagen lattice. The samples were observed under an inverted Olympus microscope (Tokyo, Japan). The MIPAR Image Analysis Software (7) recipe used to quantify the images utilizes stain deconvolution approach as described elsewhere (8). For our analysis, the recipe separated the trichrome stain into grayscale representations for effective contrast. The grayscale images are then segmented to identify total tissue, excluding slide background (white space), and to isolate collagenous tissue. The collagen area fraction measurement is made relative to the total tissue area. Each group comprised of four different animals.

### Cross-Sectional Area of Cardiomyocytes

The cross-sectional cardiomyocyte area was calculated in Top2b KDN cells, as well as, in primary adult cardiomyocyte culture. For the Top2b KDN cells, the phase contrast microscopy was used to identify cells. Any cells which did not appear to have sarcomere or the shape indicative of cardiomyocyte (such as elongated) were discarded. Random area on the slides were selected and roughly 150–200 cells/slide were used to calculate area. Please note the graph represents cardiomyocytes from five different slide in each subgroup. Measurement in primary culture cardiomyocytes were done from the BAPTA preparation with exactly same rigor applied as with Top2b KDN cells.

### Top 2b Knockdown H9c2 Cell Line

H9c2 neonatal rat cardiomyocyte cell line was obtained from ATCC (Manassas, VA, USA). Top 2b knockdown was achieved by pGFP-C-shLenti transduction (OriGene Technologies, Rockville, MD USA). Four different shRNA constructs were used to generate different lentivirus. The production and the final infection of the H9c2 cells were carried out as per manufacturer's protocol. Infected cells were selected with puromycin. The same steps were carried out with scrambled shRNA also.

### MTT Assay

MTT (3-[4,5-dimethylthiazol-2-yl]-2,5-diphenyltetrazolium bromide) assays were carried out with the aid of a Proliferation Kit I (MTT) kit. Briefly, H9c2 cells were cultured in 96 well microplates (tissue culture grade, flat-bottom). MTT was added directly in the cell culture buffer for 4 h with subsequent incubation with solubilization buffer overnight as per manufacturer's protocol. The 96 well plate was evaluated on an ELISA reader at 550–600 nm. Six different animals were used within each group.

### Hoescht 33342 Staining

Plated H9c2 cells were washed with Dulbecco's phosphate-buffered saline (DPBS). Enough volume of Hoechst 33342 [purchased from Sigma-Aldrich (Darmstadt, Germany)] working solution (10 μg/mL in DPBS) was added to the plated cells to completely cover the sample. Thereafter, the samples were incubated for 10 min in a dark room. Samples were then washed with DPBS to remove excess stain. The cells were visualized under fluorescent microscope after the samples were treated with anti-fade mounting medium. The samples were analyzed with Image J software. The size and the intensity of the fluorescence was reported as corrected total nuclear fluorescence which was calculated by Integrated Density—(Area of selected cell × Mean fluorescence of background readings). *N* = 4 within each group.

### Senescence Associated Beta-Galactosidase Staining

The senescence associated beta-galactosidase staining (Cell Signaling Technology, Danvers, MA, USA) were performed as per manufacturer's instructions. The results were reported as the fraction of cells stained positive with SA-beta Gal staining to the total population. *N* = 4 within each group.

### Telomere Length

Telomere length was measured by flow cytometry. The samples were prepared as per manufacturer's instruction (Telomere PNA Kit/FITC for Flow Cytometry, Dako/Agilent, Glostrup Denmark). Briefly, the cell suspension containing the experimental and control cell line underwent DNA denaturation process for 10 min at 82°C in a microcentrifuge tube either in the presence of hybridization solution without probe or in hybridization solution containing fluorescein-conjugated PNA telomere probe. The samples were stored in a dark room overnight which was followed by post-hybridization wash and resuspension in appropriate buffer for final flow cytometric analysis. The telomere length was reported as percentage of telomeres/chromosome when compared to standardized HEK 293T cells. *N* = 3 within each group.

### Telomerase Activity

The telomerase activity was measured with TRAPeze® Telomerase Detection Kit (Millipore Sigma, Burlington MA, USA). Briefly, the kit is a gel-based system which detects the telomerase activity in a two-step process. In the first step of the reaction, telomerase adds an AG plus a telomeric repeats (GGTTAG) onto the 3′ end of a substrate oligonucleotide. In the second step, the extended products are amplified by PCR. This technique is used to measure the telomerase activity as the intensity of the PCR product directly correlates to the intrinsic telomerase activity. *N* = 3 within each group.

### Western Blot

Cells were lysed with RIPA buffer (25 mM Tris.HCl pH 7.6, 150 mM NaCl, 1% Triton X-100, 1% sodium deoxycholate, and 0.1% SDS). The cells were boiled for 5 min at 100°C. Samples were subjected to 4–15% SDS-PAGE and transferred onto PVDF membrane (catalog IPVH00010, Millipore, Burlington, MA, USA). Blocking was performed in 3% BSA and membranes were incubated in primary antibodies overnight at 4°C. Membranes were incubated with HRP-conjugated secondary antibody for 2 h at room temperature and protein was visualized using SuperSignal West Pico Luminol Enhancer Solution (product number 1859675, Thermo Scientific, Waltham, MA, USA). The bands were analyzed with Image J software. *N* = 3 within each group.

### Primary Mouse Cardiomyocyte Culture

Primary adult cardiomyocyte isolating kit by Cellutron technologies (Baltimore, MD, USA) were used to create primary cultures. α-MHC-MerCreMer-Top2b^flox/flox^ or α-MHC-MerCreMer Top2b^+/+^ and α-MHC-MerCreMer Top2b^+/flox^ mice were treated with Tamoxifen. The heart underwent perfusion after 3 months. The heart was isolated and a Langendorff perfusion under constant flow was performed as per the manufacturer's instructions. The isolated cardiomyocytes were cultured and used for experimentation after 24 h of stabilization. *N* = 5 within each group. The primary culture cells were plated in the patented AS medium from Cellutron Technologies (Baltimore, MD, USA).

### BAPTA

BAPTA/AM (1,2-bis(o-Aminophenoxy)ethane-N,N,N′,N′-tetraacetic Acid Tetra(acetoxymethyl) Ester) was purchased from Sigma-Aldrich (Darmstadt, Germany). The primary cell culture cells were washed in calcium and magnesium free phosphate-saline buffer three times. The cells were then incubated with BAPTA/AM at 10 μM concentration for 5 min and washed again with calcium and magnesium free phosphate-saline buffer three times. Imaging was done with Olympus fluorescent microscope and analyzed with Image J software. The corrected total cell fluorescence was calculated as described before. *N* = 3 within each group.

### Reverse Phase Protein Analysis

Reverse phase protein analysis (RPPA) is a high-throughput system which essentially is a microarray for the proteins. Briefly, cellular proteins were denatured by 1% SDS (with Beta-mercaptoethanol) and diluted in five 2-fold serial dilutions in dilution lysis buffer. Serial diluted lysates were arrayed on nitrocellulose-coated slides (Grace Bio Lab, Bend, OR, USA) by Aushon 2470 Arrayer (Aushon BioSystems, Billerica, MA, USA). A total of 5,808 array spots were arranged on each slide including the spots corresponding to serial diluted: (1) “Standard Lysates”; (2) positive and negative controls prepared from mixed cell lysates or dilution buffer, respectively. Each slide was probed with a validated primary antibody plus a biotin-conjugated secondary antibody. Only antibodies with a Pearson correlation coefficient between RPPA and western blotting of >0.7 were used for RPPA. The signal obtained was amplified using a Dako Cytomation–Catalyzed system (Agilent, Santa Clara, CA, USA) and visualized by DAB colorimetric reaction. The slides were scanned, analyzed, and quantified using a customized-software to generate spot intensity. Each dilution curve was fitted with a logistic model (“Supercurve Fitting” developed by the Department of Bioinformatics and Computational Biology in MD Anderson Cancer Center, “http://bioinformatics.mdanderson.org/OOMPA”). This fits a single curve using all the samples (i.e., dilution series) on a slide with the signal intensity as the response variable and the dilution steps as independent variables. The fitted curve is plotted with the signal intensities—both observed and fitted—on the *y*-axis and the log2—concentration of proteins on the *x*-axis for diagnostic purposes. The protein concentrations of each set of slides were then normalized for protein loading. Correction factor were calculated by (1) median-centering across samples of all antibody experiments; and (2) median-centering across antibodies for each sample.

### Ingenuity Pathway Analysis

Ingenuity pathway analysis (IPA) is an all-in-one, web-based software application that enables analysis, integration, and understanding of data derived from gene expression, miRNA, and SNP microarrays, as well as metabolomics, proteomics, and RNAseq experiments. IPA was generated by manually curating scientific manuscript and thereby creating a structural lattice of different mechanisms, proteins, pathways and others connected together. We took the RPPA dataset and analyzed with IPA to identify the key upstream regulator that can cause the directional change in various proteins identified with RPPA. As a result two pathways were identified (as noted in the manuscript) involving p53 and PTEN. The latter was unchanged but on closer analysis of PTEN pathway, Akt was found to be inhibited in our Western Blots.

### Human Sample

The human samples were acquired from Duke Human Heart Repository managed by Dr. Bowles. The samples were endomyocardial biopsy or explanted hearts obtained during the clinical investigations. The samples were frozen at reception and the proteins were extracted from these frozen samples. The transfer of the biological samples followed the institutional protocol and was received under Material Transfer Agreement (MTA). *N* = 5 within each group. Please note no identifiable human data was transferred in the process as per MTA agreement.

### Statistical Analysis

The comparison between two groups were analyzed with student *t*-test with Bonferroni correction. Multiple groups were compared with one-way ANOVA. The number of samples used for each figures has been identified in the legend.

## Results

We established a conditional, inducible and site-directed genetic mouse model with cardiomyocyte-specific deletion of Top2b (α-MHC-MerCreMer-Top2b^flox/flox^ mice). We achieved cardiomyocyte-specific Cre induction by treating mice with tamoxifen (25 mg/kg of body weight by gavage, once a day for 5 consecutive days) as previously described (6). After 1 and 3 months from the Top2b^−/−^ in the mice heart, we examined the cardiac function.

**Figure 1:**
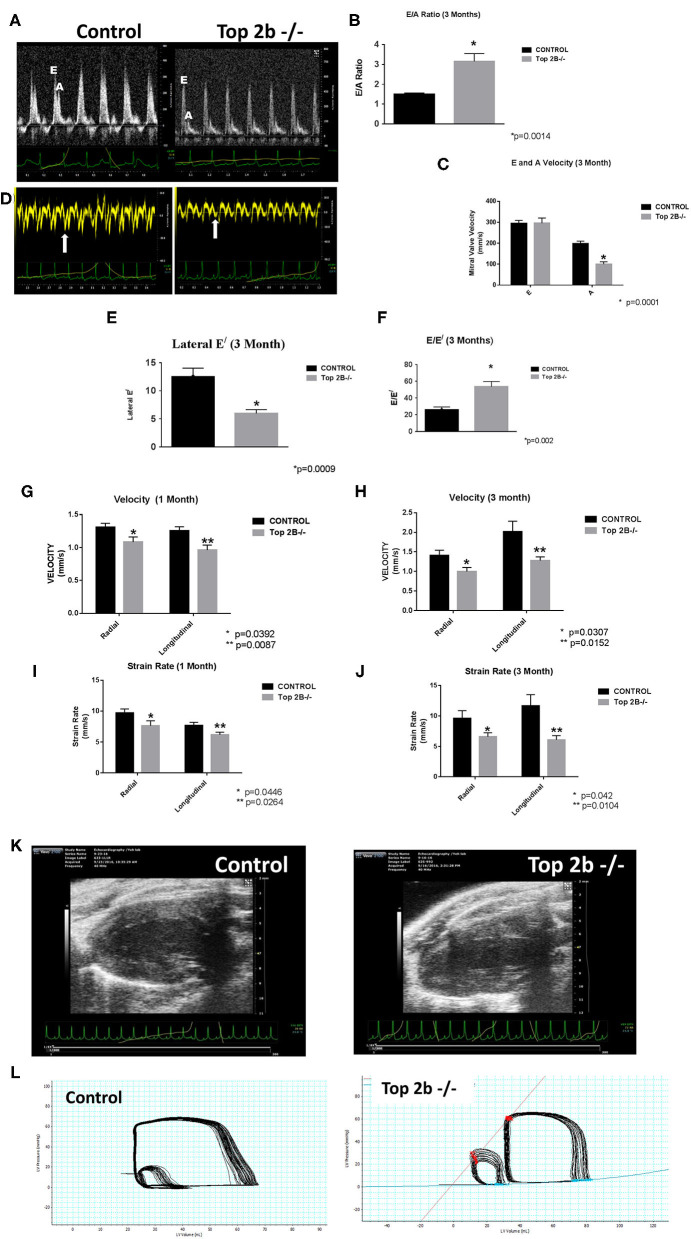
Functional Analysis show that Top2b^−/−^ mice develop diastolic dysfunction. **(A)** The echocardiographic images of pulse wave Doppler flow through the mitral valve inflow measuring E (early diastole) and A (atrial kick) velocities. The E/A ratio is increased in Top 2b^−/−^ mice compared to control. **(B)** Graphical analysis shows a significant increase E/A ratio at 3 months (*p* = 0.014). **(C)** The major cause of increase is primarily due to significant decrease in A velocity through the mitral inflow valve (*p* = 0.001). **(D)** The echocardiographic images of the tissue Doppler Imaging of the lateral wall showing a significant decrease in excursion of the lateral wall in Top 2b^−/−^ mice compared to control. **(E)** Graphical analysis shows that's E^/^ of the lateral wall is significantly decreased (*p* = 0.0009) which results in **(F)** increased E/E^/^ ratio (*p* = 0.002). **(G)** Strain analysis show that radial (*p* = 0.0392) and longitudinal velocities (*p* = 0.0087) were significantly decreased in Top 2b^−/−^ mice respectively at 1 month which prevailed at **(H)** 3 months also [radial velocity (*p* = 0.0307); longitudinal velocity (*p* = 0.0152)]. **(I)** The strain rate was also significantly decreased in Top 2b^−/−^ mice both radially (*p* = 0.0446) and longitudinally (*p* = 0.0264) at 1 month. **(J)** The significant decrease in strain rate persisted at 3 months also [radially *p* = 0.042; longitudinally (*p* = 0.0104)]. **(K)** Parasternal long axis view of the left myocardium in control and Top 2b^−/−^ mice. There is an increase in thickness in the posterior and septal wall. Posterior wall was significantly thick in Top 2b^−/−^ (1.25 mm) when compared to control (0.85 mm), however there was no significant change in interventricular septum (Supplementary Table 1). (Please note the video files are available online). **(L)** The pressure–volume tracings generated with high fidelity LV catheters. The smaller loops represent IVC obstruction while the big loops are the normal tracings.

Echocardiogram showed evidence of possible diastolic dysfunction as measured by an increase in E/A (Figures 1A, B). The predominant cause seemed to be a decrease pressure differential due to atrial kick (Figure 1C). Additionally, the lateral E^/^ was also decreased (Figures 1D,E) resulting in increased E/E^/^ (Figure 1F) thus culminating in echocardiographic features indicative of diastolic dysfunction. Furthermore, we performed strain analysis. There was a decrease in longitudinal and radial strain velocity after 1 month from Top 2b^−/−^ (Figure 1G) which persisted at 3 months (Figure 1H). The strain rate was also decreased longitudinally and radially during the same time period (Figures 1I,J). Structurally, there was an increase in the posterior wall thickness in diastole, as well as, in the relative wall thickness (Figure 1K and Supplementary Video). However, there were no significant changes in LV mass (Supplementary Table 1). Most importantly, there were no changes in EF, heart rate, lung weight, tibial length, and body surface area between Top2b^−/−^ mice when compared to the control cohort (Supplementary Table 1).

To further characterize the diastolic dysfunction, we performed PV loops. A high fidelity Millar catheter was inserted in the left ventricle through apical puncture. PV loops were generated and changes in the PV relationship was assessed (Figure 1L). At 3 months, there was a significant decrease in minimum *dp*/*dt* with no change in maximum *dp*/*dt* in Top2b^−/−^ mice when compared to control (Table 1). This indicates a decrease in left ventricular relaxation with no change in contractility. Furthermore, the Tau index (time required by ventricles to relax) was increased in Top2b^−/−^ mice indicating impaired diastolic function (Table 1). There was no change in EF, stroke work, or cardiac output (Table 1).

**Table 1:** Pressure–volume measurements in control and Top 2b deleted mice at 3 months (*n* = 4).

**Pressure-volume**	**Control**	**Top 2 B^**−/−**^**	***p*-Value**
**loop parameters**	**(Mean ± SEM)**	**(Mean ± SEM)**	
Cardiac output (mL/min)	18.78 ± 1.48	17.50 ± 0.48	NS
Stroke volume (μL)	40.22 ± 2.45	39.53 ± 0.91	NS
Heart rate (bpm)	462.5 ± 13.80	439.0 ± 16.16	NS
Ejection fraction (%)	55.14 ± 2.04	54.48 ± 1.41	NS
Positive dp/dt (mm Hg s^−1^)	5029.25 ± 571.74	5282.50 ± 201.72	NS
Negative dp/dt (mm Hg s^−1^)	4932.25 ± 412.07	3423.00 ± 121.71	0.0126
Tau (ms)	10.48 ± 1.22	13.94 ± 0.43	0.0365
*R*-square (correlation coefficient)	0.99 ± 0.01	0.24 ± 0.05	<0.0001

Perturbation with inferior vena cava (IVC) occlusion showed an attenuated response with respect to end diastolic volume change to a given PV area in Top2b^−/−^ mice (Supplementary Figure 1) when compared to controls signifying decreased relaxation [Correlation Coefficient [*R*^2^] PV area vs. end-diastolic volume (0.99 ± 0.01 in control vs. 0.24 ± 0.05)] indicating compromised ventricular capacitance [defined as changes in end-diastolic pressure–volume relationship (EDPVR), i.e., leftward/upward shift in EDPVR indicate decreased ventricular capacitance] (Table 1) (9, 10).

**Figure 2:**
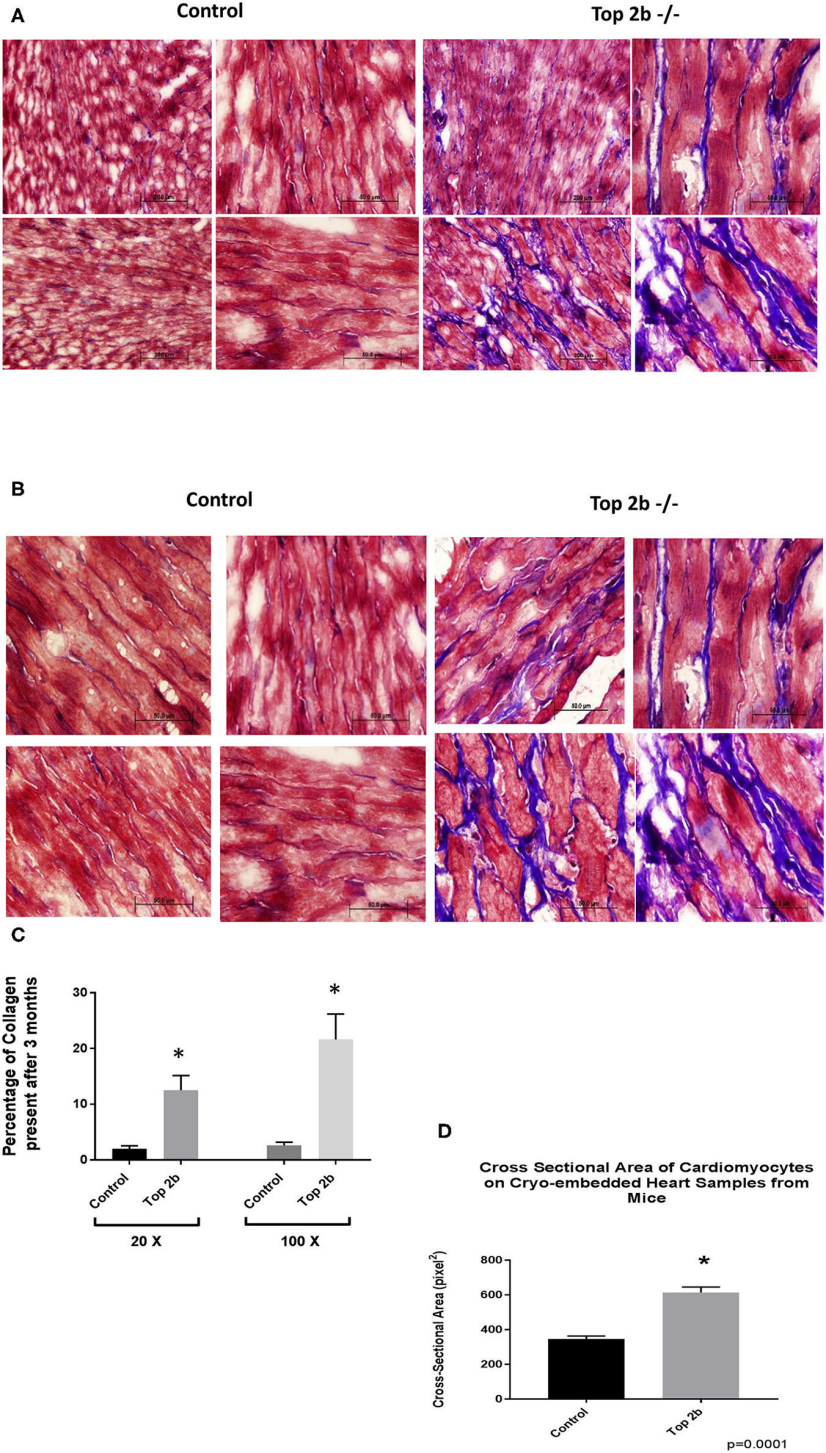
Histological Analysis show that Top2b^−/−^ mice develop diastolic dysfunction. **(A)** Histological analysis was carried with Trichrome staining with Weigert's Iron Hematoxylin for nuclear staining, Biebrich Scarlet/Acid Fuchsin Solution for cytoplasmic staining and aniline blue for collagen lattice. Slides at 20× **(A)** and 100× **(B)** show significant collagen deposition in Top 2b mice when compared to age-matched controls. **(C)** The quantitative analysis was carried out with MIPAR software using collagen recipe showing morphometric increase in collagen in Top 2b^−/−^ mice when compared to its control counterpart (*p* < 0.001). Additionally, the cross-sectional area of cardiomyocytes showed increase in size compared to control hearts **(D)**. Image J software was used to calculate pixels present in the area. The measurement was carried out by a blinded investigator.

Histological analysis of the cardiac tissues was performed. A trichrome stain was carried out with red identifying the cardiomyocyte, black signifying the nucleus while blue staining representing collagen. At 3 months, there was a significant deposition of collagen in Top2b^−/−^ mice Figure 2A (20×) and Figure 2B (100×). Quantitative analysis showed an increased total extracellular volume (Figure 2C). Interestingly, the cardiomyocyte volume was also increased (Figure 2D); a feature of diastolic dysfunction as identified by Douglas and Tallant (11).

**Figure 3:**
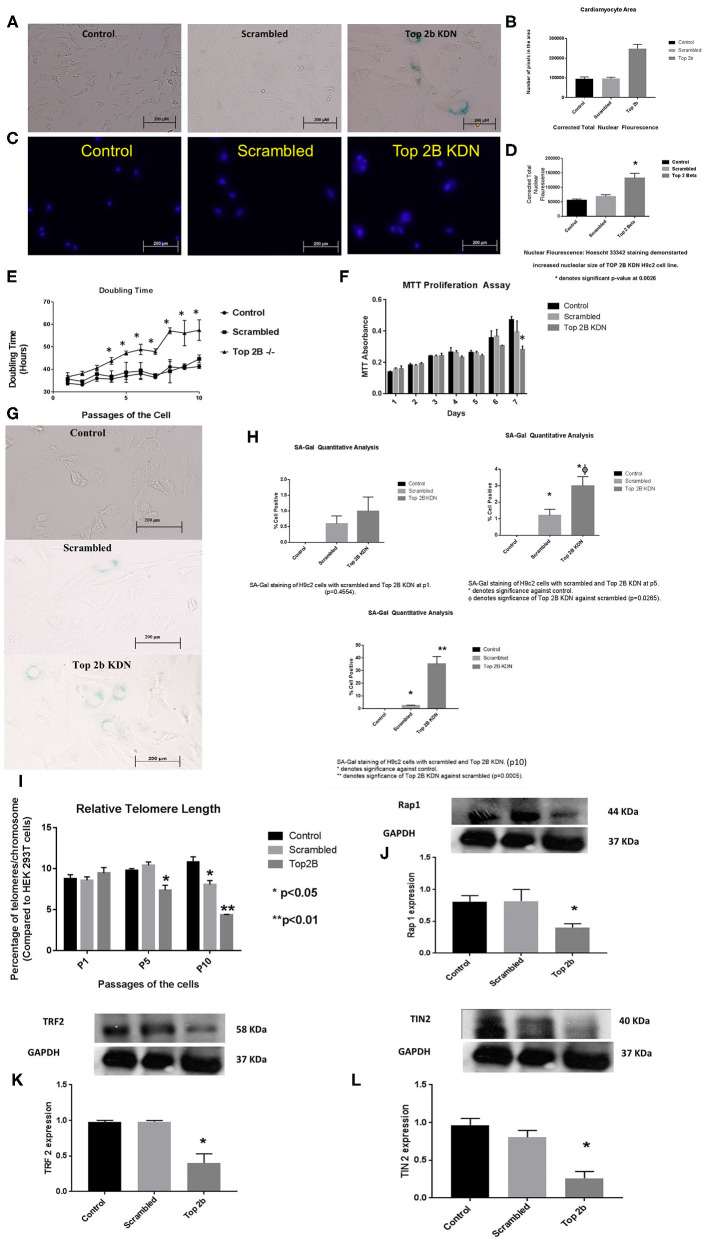
Top 2b KDN H9c2 cells show cellular, sub-cellular and molecular features of diastolic dysfunction. The features are more pronounced with aging. **(A)** Phase contrast images (at 20×) shows increase in cell size after Top 2b KDN in H9c2 cells, a feature found in diastolic dysfunctional cardiomyocytes. **(B)** The graphical analysis shows a significant increase in size as calculated by pixel area measured by Image J software. **(C)** Fluorescent microscopy shows an increase in nuclear size which is an indication of a non-dividing cell, marked for aging. **(D)** Graphical analysis shows correct total nuclear fluorescence which takes into account the intensity of fluorescence and size of the nucleus. The corrected total nuclear fluorescence was significantly increased in Top 2b KDN cell line. **(E)** Graphical representation of doubling time for the H9c2 cells show that Top 2b KDN results in slowing of the growth rate. The analyses were done when the cells reached 70% confluency. **(F)** A decrease in proliferation was also confirmed with MTT assay which shows decrease in proliferation at day 7 in Top 2b KDN H9c2 cell line. **(G)** Phase contrast microscopy showed increased staining of Senescence associated beta-galactosidase staining indicating cells marked for senescence or aging. **(H)** The senescence was progressive as graphical analysis of SA-Beta-gal staining shows temporal increase in staining from passage 1 (p1), to p5 with eventually ~ 37% of cells in Top 2 KDN H9c2 cell line marked for senescence at p10 which was statistically significant. **(I)** Percentage of telomeres/chromosomes were measured against HEK 293T cells. Top 2 b KDN shows a significant progressive decrease with passaging of the cells. The telomere length of scrambled was also decreased at p10 as selection with puromycin may have induced premature aging. However, Top 2b KDN cells showed significant decrease in telomere length when compared to both control and scrambled at p10. **(J)** The decrease in telomere length was due to a significant decrease in components of shelterin complex namely Rap1, **(K)** TRF2 and **(L)** TIN2. Quantitative analysis of each Western Blot is present below the blots.

To further characterize the role of Top2b in cardiomyocyte, we generated H9c2 cell line with Top2b knockdown (Top2b KDN). This was achieved with lentivirus infection of siRNA against Top2b. Top2b KDN resulted in an increase in cardiomyocyte volume, a feature identified in the histological analysis as mentioned above (Figures 3A,B). Additionally, the nuclear size was also increased as measured by Hoescht 33342 fluorescence (Figures 3C,D). Interestingly, successive splitting of cells resulted in progressive decrease in the doubling time with subsequent passages (Figure 3E). This is very intriguing as it is well-established that diastolic dysfunction in humans worsens with passing time (12, 13). This was also evident with MTT assay which showed slow growth at day 7 in H9c2 cell line with Top2b KDN when compared to its scrambled counterpart (Figure 3F). To further characterize, if the decrease in the proliferation of H9c2 cells carrying Top2b KDN was due to accelerated cellular senescence, we performed senescence associated beta galactosidase (SA Beta Gal) staining (Figure 3G). The SA Beta Gal positive staining increased progressively with subsequent passaging (Figure 3H). Furthermore, at passage 10, roughly 40% of the Top2b KDN showed SA Beta Gal staining indicative of cellular senescence (Figure 3H).

To further delineate the pathways behind slow growth of the cardiomyocyte cell line and development of diastolic features, we examined the telomere lengths in these cell lines. We found that relative telomere length was significantly decreased when compared to the scrambled as well as the control cell with successive passaging (Figure 3I). We examined the shelterin complex, an aggregate of six proteins that protect chromosomal ends. It appeared that telomere length decrease was primarily due to compromised shelterin complex as RAP-1 (Figure 3J), TRF2 (Figure 3K) and TIN2 (Figure 3L) was decreased. However, other components of shelterin complex such as POT1 (Supplementary Figure 2A), TRF1 (Supplementary Figure 2B) and TPP1 (Supplementary Figure 2C) remained unchanged. There was no change in telomerase activity in these cell lines also (Supplementary Figure 3). Furthermore, the retinoblastoma protein was also dephosphorylated indicating that the cells were committed to cellular senescence (Supplementary Figure 2D).

**Figure 4:**
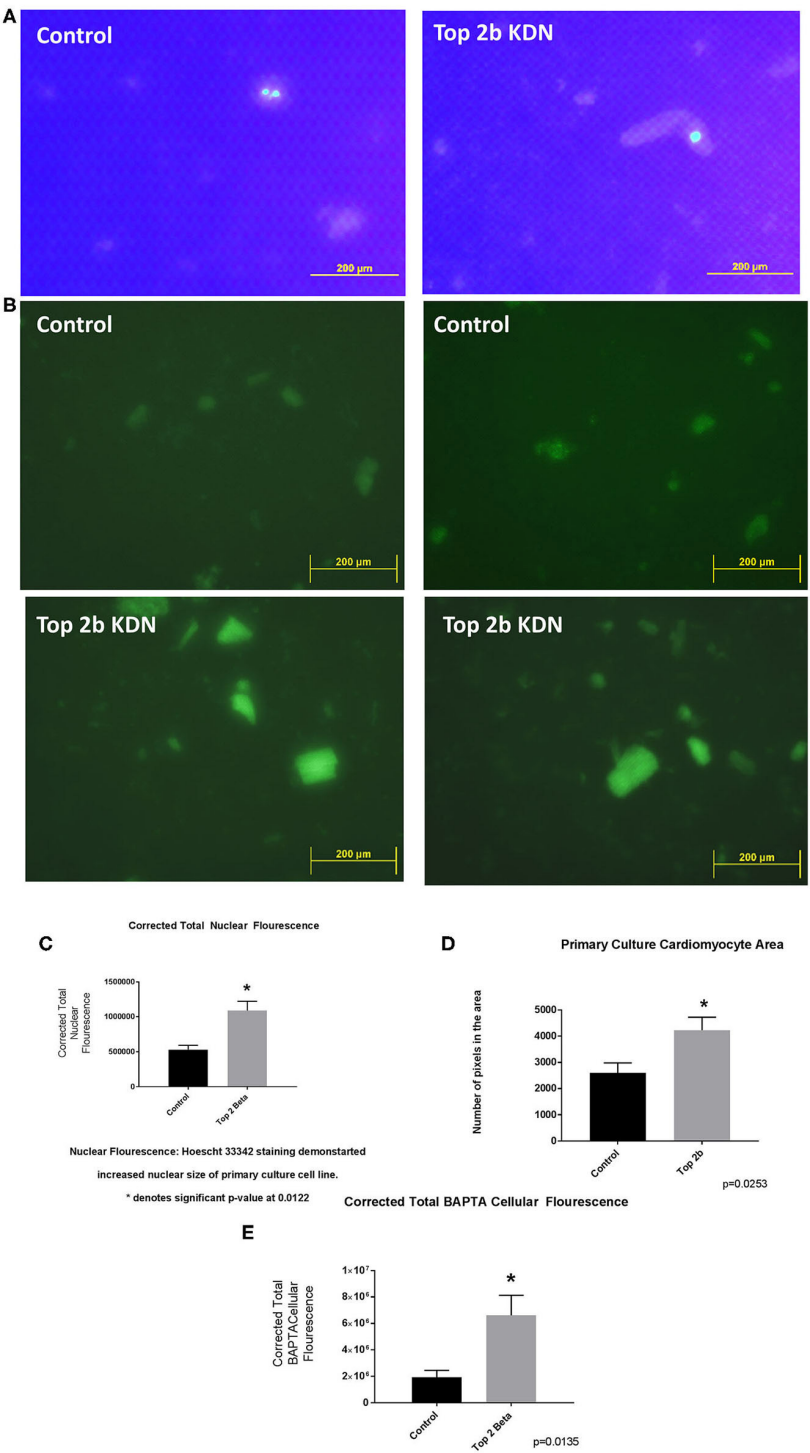
Top 2 b^−/−^ primary culture cell line show cellular, sub-cellular and molecular features of diastolic dysfunction. **(A)** The nuclear size was found to be increased in Top 2b^−/−^ primary cell culture line when compared to the control. **(B)** BAPTA staining of the primary culture cell line shows significant increase in cytoplasmic calcium which is indicative of a calcium leak, a phenomenon that is present in diastolic dysfunction. **(C)** Graphical analysis shows significant increase in corrected total nuclear fluorescence in Top 2b^−/−^ primary culture cell line. **(D)** The size of cardiomyocytes in primary culture cell line was also significantly increased in Top 2b^−/−^ conforming to previous experiments. **(E)** Graphical representation of corrected total BAPTA fluorescence shows significant increase in BAPTA fluorescence in Top 2b^−/−^ primary culture cell line.

To further validate the cell line findings, we established a primary cardiomyocytes culture line from our mouse model. We identified increased nuclear size and fluorescence congruent to our H9c2 Top2b KDN cell line (Figures 4A,C). Additionally, the cardiomyocyte volume was increased akin to the established cell line (Figure 4D). To further characterize if primary cell cultures cardiomyocytes indeed have cellular features of diastolic dysfunction, we looked at the calcium signaling. We incubated the cells with BAPTA-AM to visualize the intracellular calcium load. BAPTA-AM showed significant increase in fluorescence in primary culture cardiomyocytes with Top2b deletion (Figures 4B, E). This is congruent to the features identified in human diastolic dysfunction as there is increased intracellular calcium due to calcium leak (3). Therefore, the cellular changes noted in our Top2b cell line and primary culture cardiomyocytes showed cellular changes in keeping with diastolic dysfunction in the human heart.

**Figure 5:**
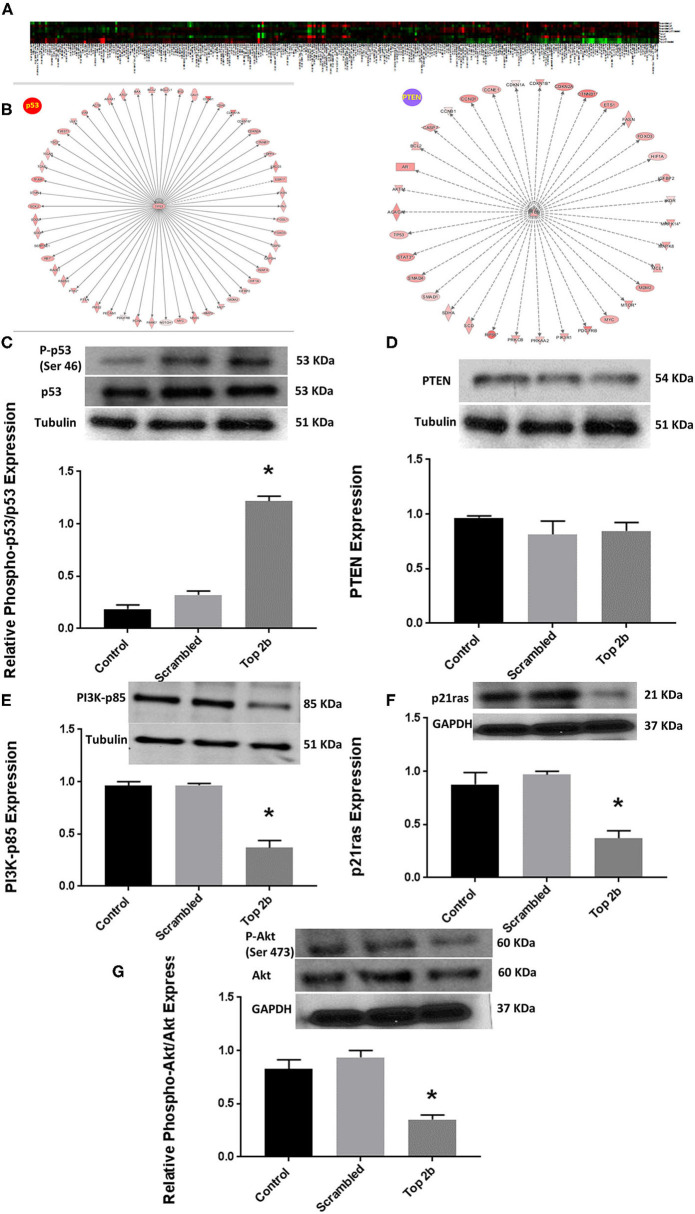
Top 2b decrease mediated diastolic dysfunction is associated with activation of p53 and inhibition of Akt phosphorylation. **(A)** Heat map generated by reverse phase protein analysis. The full list of the protein is in Supplementary Table 2. **(B)** Ingenuity Pathway Analysis with identification of upstream regulator resulted in significant identification of p53 and PTEN as putative signaling mechanism that may be involved in diastolic dysfunction (A larger version of the figure is available in Supplementary Figure 5). **(C)** Top 2b KDN H9c2 cell line showed a significant increase in phospho-p53/p53 expression. **(D)** However, there was no change in PTEN expression. **(E)** The upstream regulator PI3K-p85 was significantly decreased which was primarily due to significant decrease in p21ras **(F)**. **(G)** Conversely, the phospho-Akt expression was significantly decreased (*p < 0.001).

To further elucidate the molecular mechanism precipitating Top2b mediated diastolic dysfunction, we performed reverse phase protein arrays (RPPA). Briefly, the cell line carrying Top2b KDN were used to generate the array. Out of 526 proteins examined, 304 showed changes from its scrambled counterpart (Figure 5A, Supplementary Table 2). We validated the changes in the protein identified with RPPA reflected in our samples (Supplementary Figure 4). We used the array data and identified the upstream regulator with the use of IPA. A significant match was achieved and identified the tumor suppressor gene p53 and PTEN (Figure 5B; a larger version of the figure is available in Supplementary Figure 5). We performed Western blots and showed that indeed phosphorylation of p53 was increased (Figure 5C), however, no change in PTEN was identified (Figure 5D). The upstream substrate of PTEN, PI3K-p85 was significantly decreased (Figure 3E) which we showed was due to a decrease in p21ras (Figure 5F). Furthermore, there was a significant decrease in cell survival signaling, namely Akt in the Top2b KDN cell line (Figure 5G). We showed that phosphorylation of p53 was increased, albeit Akt phosphorylation was decreased in our primary cardiomyocyte cultures also. This suggests that the Top2b decrease is associated with attenuated cell survival signaling of Akt and increase in cellular senescence with possible activation of p53; novel mechanisms identified in possible Top2b mediated diastolic dysfunction.

**Figure 6:**
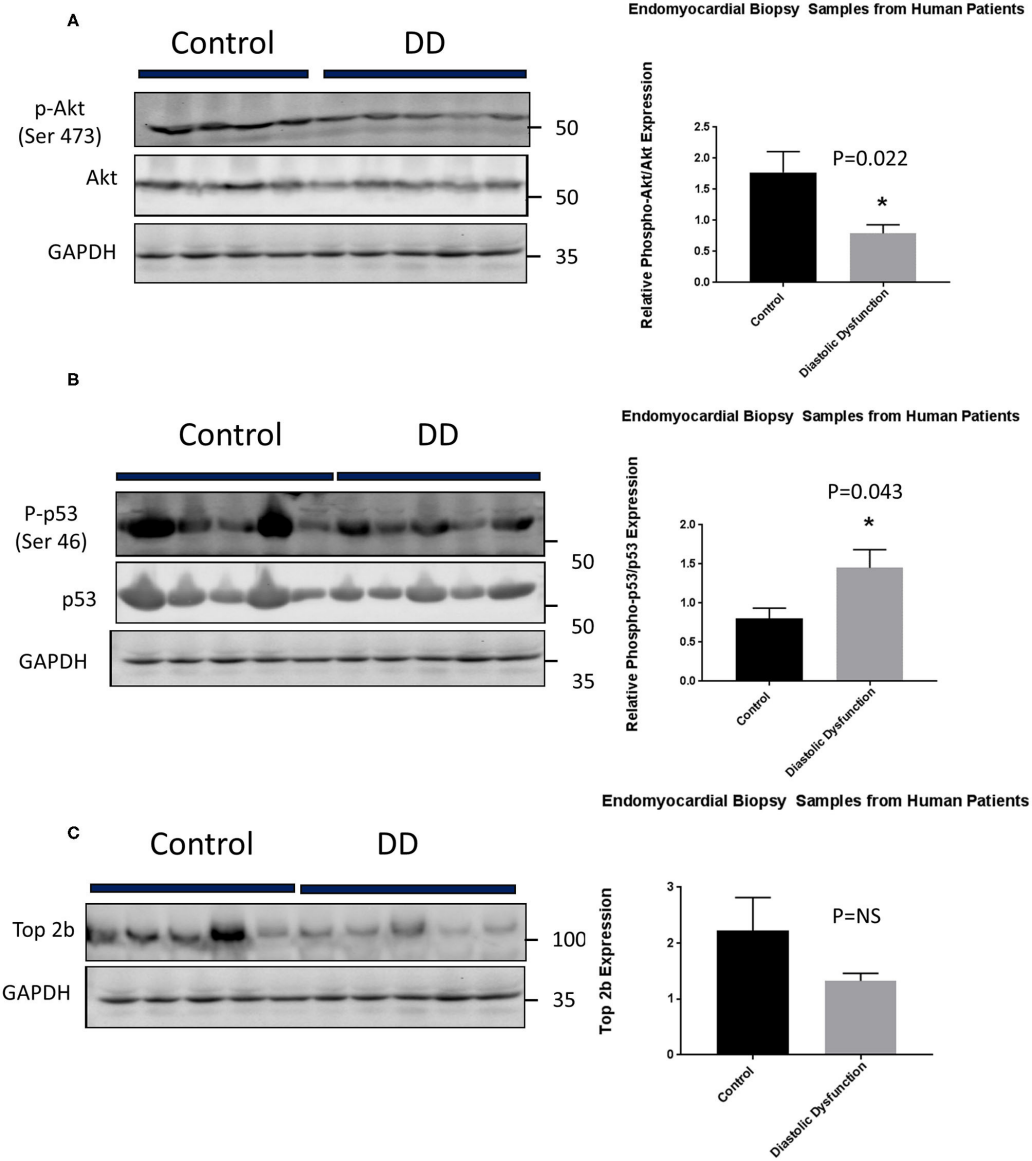
Top 2b decrease mediated diastolic dysfunction is primarily due to activation of p53 and inhibition of Akt phosphorylation in human hearts. **(A)** Phospho-akt/Akt samples showed a significant decrease in phosphorylation of Akt when compared to control (*p* = 0.022). Conversely, **(B)** phospho-p53 was significantly increased in diastolic dysfunction patients (*p* = 0.043). However, there was no statistical significance in **(C)** Top2b expression between patients with diastolic dysfunction and control samples.

Lastly, we embarked on confirming our findings in human population. We obtained human heart biopsy samples from Duke University Human Heart Repository. The baseline characteristics of the patients are represented in Supplementary Table 3. Western Blot analysis was performed to assess for Top2b, phospho-Akt, Akt, phospho-p53, and p53. The analysis showed that there was a significant decrease in phosphorylation of Akt. Conversely, the phosphorylation of cell senescence with activated p53 as increased. There was statistical non-significant decrease in topoisomerase 2 beta expression (Figure 6).

## Discussion

Diastolic dysfunction has been considered a holy grail for cardiac research as the precise mechanism and treatment option remains an enigma. There have been numerous clinical trials such as I-PERSEVE, CHARM-Preserved, DIG-ancillary trial, SENIORS, and PEP-CHF which have been negative (5). The reason for failed clinical trials stem from the use of medications beneficial for reduced EF in a population with preserved EF. Additionally, there are no discernable and widely accepted preclinical models to study diastolic dysfunction (14). We propose a first genetic model of Top2b heart-specific deletion which resulted in findings akin to human diastolic dysfunction to some extent. Most importantly, we showed for the first time that these changes can be a function of time.

Top2b is an enzyme that regulates the overwinding or underwinding of DNA during cellular processes such as replication, transcription, and chromosomal segregation by altering DNA topology (15–17). These functions are mediated by double stranded breaks which are re-annealed following the completion of cellular task. Phylogentically, topoisomerase existed in only one isoform (18). However, more recently in the animal kingdom, it has diverged into Top2a (alpha), and Top2b (18), the former being more prevalent in the dividing cells while the latter is present in the quiescent cells (19–21). Therefore, it is not surprising that over the years, Top2b has been implicated in more cellular processes besides its role in DSBs for cellular replication (19–24). This includes but is not limited to immediate early-gene expression (22–24), gene repression (25, 26), phosphorylation (27, 28) as well as SUMOylation (29–31). Hence, Top2b is emerging as a multifaceted protein.

Initial studies looking at the differential expression of Top2a and Top2b identified that Top2b may be related to aging. This was identified by Kondapi and colleagues (32, 33) who found decreased expression of Top2b in whole brain preparation of an adult rat when compared to its younger counterpart. More specifically, the expression was decreased in cerebellum, and cerebellar region. This was further confirmed in sheep neuronal cell preparation where Top2b activity was decreased in an age-dependent manner (34). Despite these publications, the precise role of Top2b in aging remains elusive.

Cardiovascular aging or age-associated cardiovascular diseases are characterized by a wide array of changes including, diastolic dysfunction (35). Diastolic dysfunction is comprised of impaired compliance and relaxation of ventricular chambers stemming from morphological changes that are accelerated with aging (35). Thus, the mechanism of diastolic dysfunction may lie in the process of aging and, therefore, may be inter-related with each other.

At the organ level, different structural and functional changes in the heart culminate into diastolic dysfunction. At the structural level, there is a decrease in myocyte number, and an increase in myocyte and nuclear size with myofibril disarray (36, 37). As myocytes are lost they are replaced with fibroblasts, and the remainder of the cardiomyocytes undergo hypertrophy. As the fibroblasts produce collagen, interstitial fibrosis occurs and the heart becomes stiffer and less compliant. The stiffer and less compliant ventricle results in diastolic dysfunction (38–42). Our genetic mice model follows the same morphological, structural, and functional patterns as identified in diastolic dysfunction. Most importantly, it also has the same temporal relationship as the development of diastolic dysfunction. Recent studies highlighted a “two-hit” model but it remains to be established if it follows the temporal relationship we see in our human cohort (43). In our model, we saw increased cell size, with enlarged nucleus and calcium handling noted in diastolic dysfunction. It appears that these changes may be associated with activation of p53 and inhibition of Akt.

Adult mammalian cardiomyocytes are differentiated postmitotic cells that lack proliferative capacity and re-activation of cell cycle. In fact due to hemodynamic stress the cardiomyocytes undergo hypertrophic response rather than hyperplastic response to compensate for the insult. However, the identified pathways such as p53 and Akt are crucial for cardiac well-being.

Recent studies were carried out looking at the role of p53 in initiation of heart failure. The experiments entailed looking at the trajectory of cardiomyocyte remodeling and distinct cardiomyocyte gene programs encoding morphological and functional signatures in cardiac hypertrophy and failure, by integrating single-cardiomyocyte transcriptome with cell morphology, epigenomic state, and heart function. It was identified that sustained overload stimuli induced the accumulation of oxidative DNA damage, leading to p53 signaling activation during hypertrophy. Single-cell analysis of cardiomyocyte-specific knockout mice provided strong evidence indicating that p53 in cardiomyocytes increases cell-to-cell transcriptional heterogeneity, induces morphological elongation, and drives pathogenic gene programs by disrupting the adaptive hypertrophy modules and activating the heart failure module, thereby elucidating how DNA damage accumulation leads to heart failure (44). Additionally, studies have shown that p53 activation at the G2–M phase is necessary and sufficient for cellular senescence (45, 46). Overall it could be plausible that initiation of the cellular senescence may be due to, in part, by activation of p53.

Similarly, Akt is a well-known survival signal present in the heart. Studies suggest that overexpression of Akt protects the heart from pathological hypertrophy and development of contractile dysfunction (47). Akt has been implicated in cell death, calcium cycling proteins, heart metabolism and heart failure (48). Therefore, Akt seems to be important in cardiac function.

Overall, it appears that these identified pathways may have a role to play in precipitating diastolic dysfunction. Since Top 2b does undergo change with aging, it may act as a prime regulator. However, future research is clearly warranted to establish if changes in the molecular mechanisms are the cause or effect of Top 2b decrease. Nonetheless, these mechanisms highlight potential new therapeutic targets for future pharmacological interventions.

## Limitations

This study has numerous limitations. H9c2 cell line was used in determination of role of Top 2b KDN in diastolic dysfunction. It is neonatal cardiomyocytes and as such represents a fetal phenotype. Therefore, genetic perturbation may lead to changes which are more widespread due to fetal phenotype. To counteract this confounding variable, we duplicated the experiments in primary cell culture, to provide further evidence of Top 2b decrease between different cell lines.

Additionally, we performed ingenuity analysis on RPPA from the neonatal cell line. We identified p53 and Akt as associated factors. is The RPPA experiments are hypothesis generating as future experiments with perturbation of Top 2b, Akt, and p53 are clearly warranted to delineate the cause-effect relationship between these signaling mechanism(s). Finally, the human tissue experiments were done to verify preclinical findings. Although no change in Top2b were observed the trend would support the preclinical conclusions. Certainly, more samples are required to make a firm assessment. We are also cognizant of the fact that the human sample groups are not well-matched. We were able to obtain only these samples to provide proof of concept. Nevertheless, future experimentations are clearly warranted to further explore the role of Top2b and diastolic dysfunction.

## Conclusion

We have identified that decrease in Top 2b possible results in diastolic dysfunction. At the molecular level we showed changes in Akt, cell survival signaling, and p53. It appears that the diastolic dysfunction may be related to cell survival signaling; a mechanism which requires further exploration in the context of diastolic dysfunction. This is significant as it is the first model of its kind where mice have developed diastolic dysfunction due to change in a nuclear enzyme. Furthermore, the novelty of this model lies in the fact that it showed worsening with a function of time at the cellular and subcellular levels; which is akin to human population. Overall, these studies show Top2b decrease can potentially lead to diastolic dysfunction.

## Data Availability Statement

The datasets presented in this study can be found in online repositories. The names of the repository/repositories and accession number(s) can be found in the article/Supplementary Material.

## Ethics Statement

The animal study was reviewed and approved by Institutional Review Board at MD Anderson Cancer Center.

## Author Contributions

RM conceived and designed the experiments. RM, KK, HV, WL, and AR performed the experiments. RM, GS, KK, TT, JC, KF, and J-iA analyzed and interpreted the data. RM, GS, KF, and J-iA wrote and/or edited the manuscript. All authors contributed to the article and approved the submitted version.

## Conflict of Interest

The authors declare that the research was conducted in the absence of any commercial or financial relationships that could be construed as a potential conflict of interest.

## Supplementary Material

Supplementary Table 1 Echocardiographic parameters in Control and Top 2b^−/−^ mice at 3 months. There was a significant increase in Posterior wall size, relative wall thickness, E/A and E/E/ ratio. There was no change in LV mass (echocardiographic calculation 0.8[1.04[([LVEDD + IVSd +PWd]3 - LVEDD3)]] + 0.6) or heart weight. The systolic parameters such as Ejection fraction remained unchanged. Additionally, the size of the mice, organogenesis and length were similar in two groups.

Supplementary Table 2 Baseline Characteristics of the patient myocardial biopsy sample used for the analysis. HTN: hypertension; HLD: hyperlipidemia; DM: Diabetes Mellitus; CKD: Chronic Kidney Disease; CAD: Coronary artery disease; PWT: Posterior wall thickness; IVS: Interventricular septum; LVH: Left Ventricular Hypertrophy; LVEF: Left Ventricular Ejection Fraction.

Supplementary Table 3 Reverse Phase Protein Analysis: List of tested antibodies with associated change in expression of different proteins. P1, P5 and P10 denotes the passage of cells. Scrambled denotes the control counterpart for Top 2b knockdown H9c2 cell line.

Supplementary Figure 1 Graphical representation of Pressure-volume area versus end-diastolic volume. IVC obstruction resulted in diminished response in change of EDV to a given PVA denoting reduced diastolic capacitance (16).

Supplementary Figure 2 Changes in the sheltrin complex. The other three components of shelterin complex were unchanged namely **(A)** POT1, **(B)** TRF1 as well as **(C)** TPP1. The end-effector of the senescence, **(D)** retinoblastoma protein phosphorylation was significantly decreased in Top 2b KDN H9c2 cell line indicating cells marked for senescence.

Supplementary Figure 3 The telomerase activity was unchanged in Top 2b KDN H9c2 cell lines when compared to control or scrambled. The activity is identified by PCR products generated by intrinsic telomerase activity. The arrow identifies the PCR product.

Supplementary Figure 4 Confirmation of changes in few protein identified in reverse phase protein analysis. There was an increase in JNK2, VEGFR2, and XBP-1 in Top2b KDN H9c2 cells when compared to scrambled or control. The connexin 43, CHK1, and PI3K were decreased in Top2b KDN H9c2 cells line when compared to its counterparts.

Supplementary Figure 5 Ingenuity Pathway analysis conducted on proteins from RPPA. The pictorial is the duplication of Figure 5B. The figure is enlarged for better viewing of the individual proteins.

## Abbreviations

BAPTA/AM: (1,2-bis(o-Aminophenoxy)ethane-N,N,N′,N′-tetraacetic Acid Tetra(acetoxymethyl) Ester)
DPBS: Dulbecco's phosphate-buffered saline
EDPVR: end-diastolic pressure–volume relationship
EF: ejection fraction
IVC: inferior vena cava
KDN: KNOCKDOWN
KO: knockout
MTT: (3-[4,5-dimethylthiazol-2-yl]-2,5-diphenyltetrazolium bromide)
PV: pressure–volume
RPPA: reverse phase protein assay
Top 2b: topoisomerase 2 beta.
