# Supplementary figures and images for: Topoisomerase 2B Decrease Results in Diastolic Dysfunction via p53 and Akt: A Novel Pathway
Source: Front Cardiovasc Med. 2020 Nov 6;7:594123. doi: 10.3389/fcvm.2020.594123 (PMC7709875; doi:10.3389/fcvm.2020.594123)

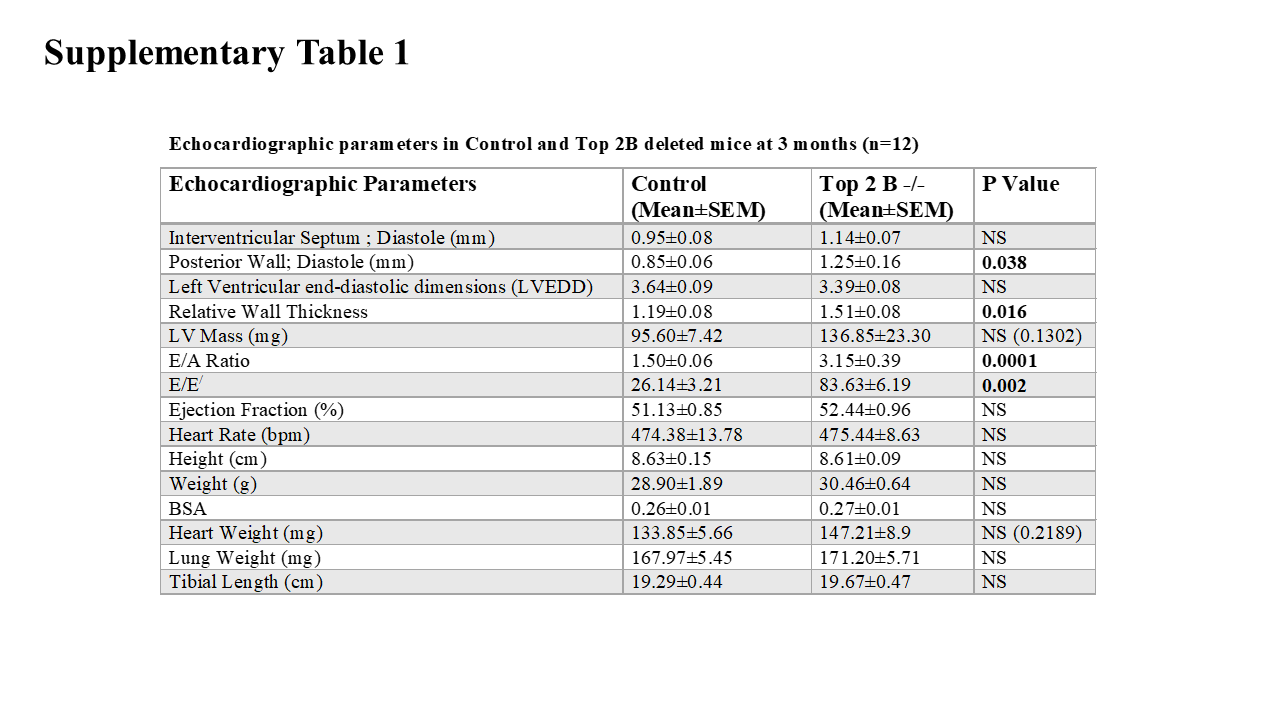

Supplement: Supplementary Table 1 — Echocardiographic parameters in Control and Top 2b−/− mice at 3 months. There was a significant increase in Posterior wall size, relative wall thickness, E/A and E/E/ ratio. There was no change in LV mass (echocardiographic calculation 0.8[1.04[([LVEDD + IVSd +PWd]3 - LVEDD3)]] + 0.6) or heart weight. The systolic parameters such as Ejection fraction remained unchanged. Additionally, the size of the mice, organogenesis and length were similar in two groups. [file Image_11.TIF]

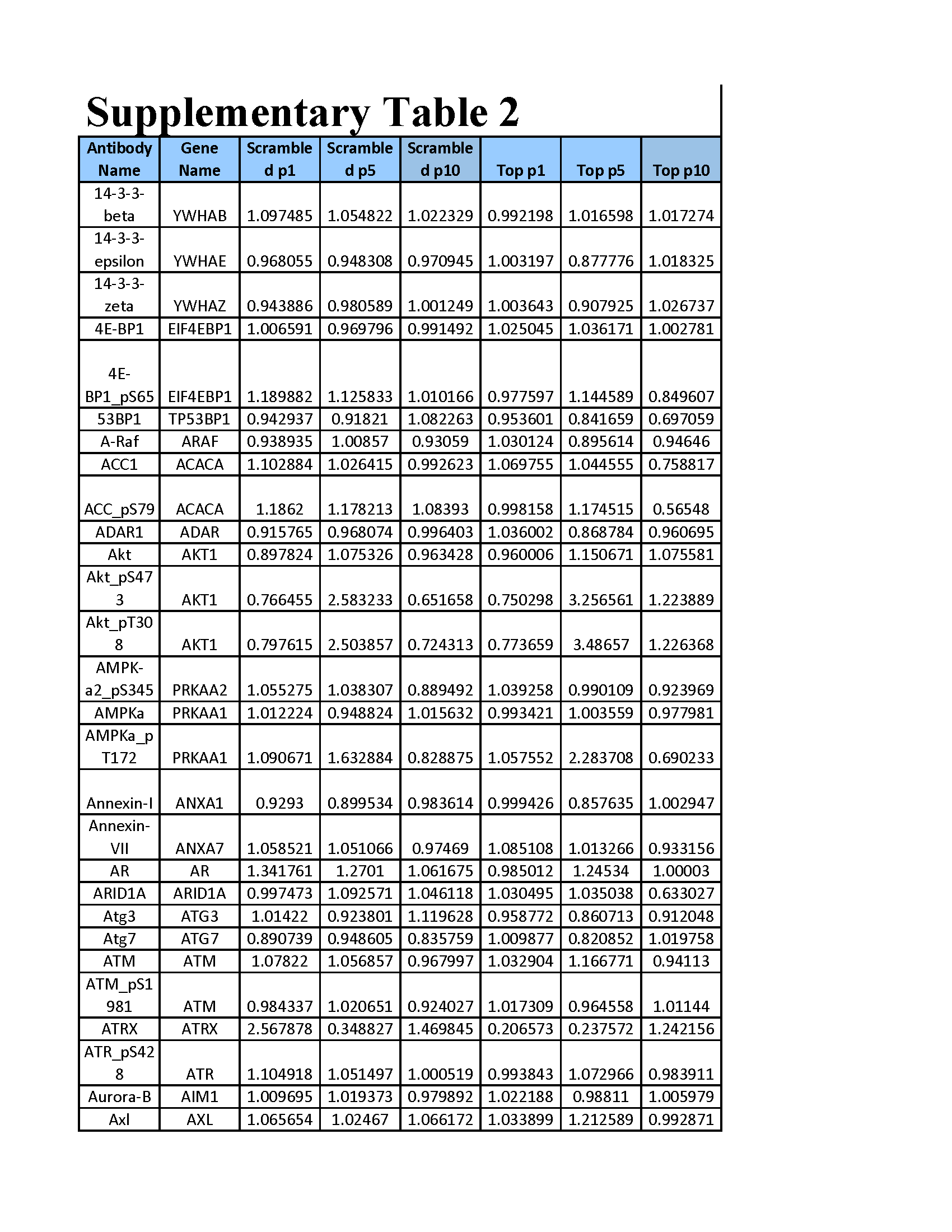

Supplement: Supplementary Table 2 — Baseline Characteristics of the patient myocardial biopsy sample used for the analysis. HTN: hypertension; HLD: hyperlipidemia; DM: Diabetes Mellitus; CKD: Chronic Kidney Disease; CAD: Coronary artery disease; PWT: Posterior wall thickness; IVS: Interventricular septum; LVH: Left Ventricular Hypertrophy; LVEF: Left Ventricular Ejection Fraction. [file Image_12.TIFF]

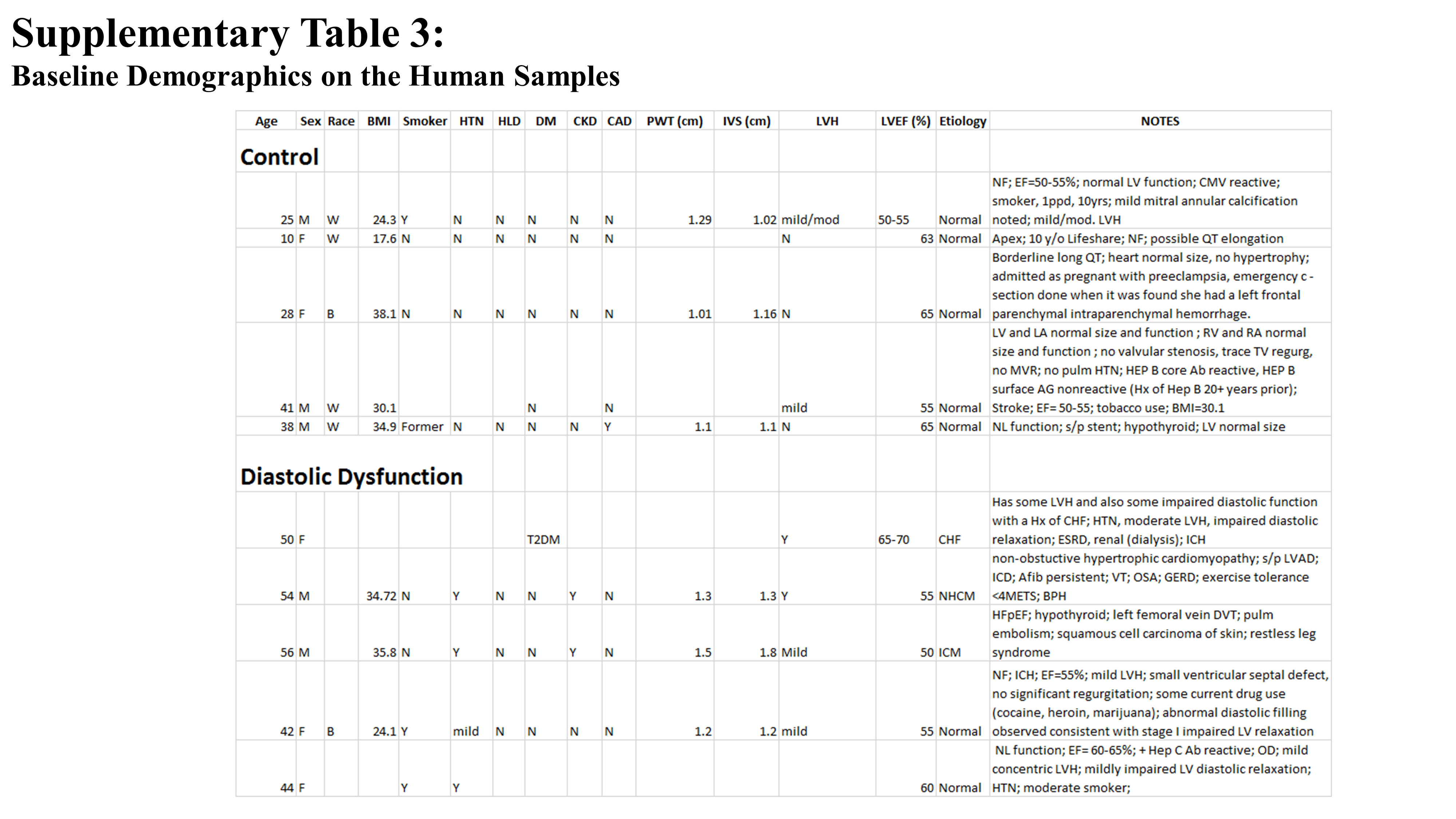

Supplement: Supplementary Table 3 — Reverse Phase Protein Analysis: List of tested antibodies with associated change in expression of different proteins. P1, P5 and P10 denotes the passage of cells. Scrambled denotes the control counterpart for Top 2b knockdown H9c2 cell line. [file Image_5.TIF]

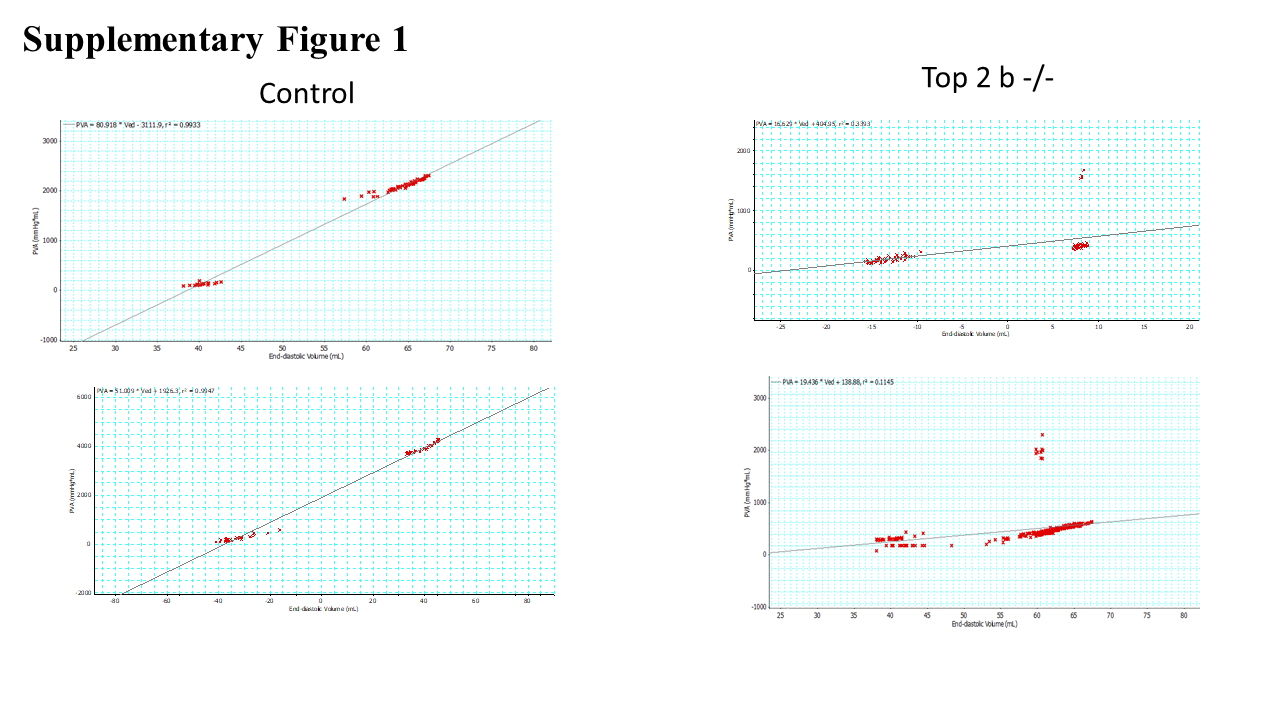

Supplement: Supplementary Figure 1 — Graphical representation of Pressure-volume area versus end-diastolic volume. IVC obstruction resulted in diminished response in change of EDV to a given PVA denoting reduced diastolic capacitance (16). [file Image_6.TIF]

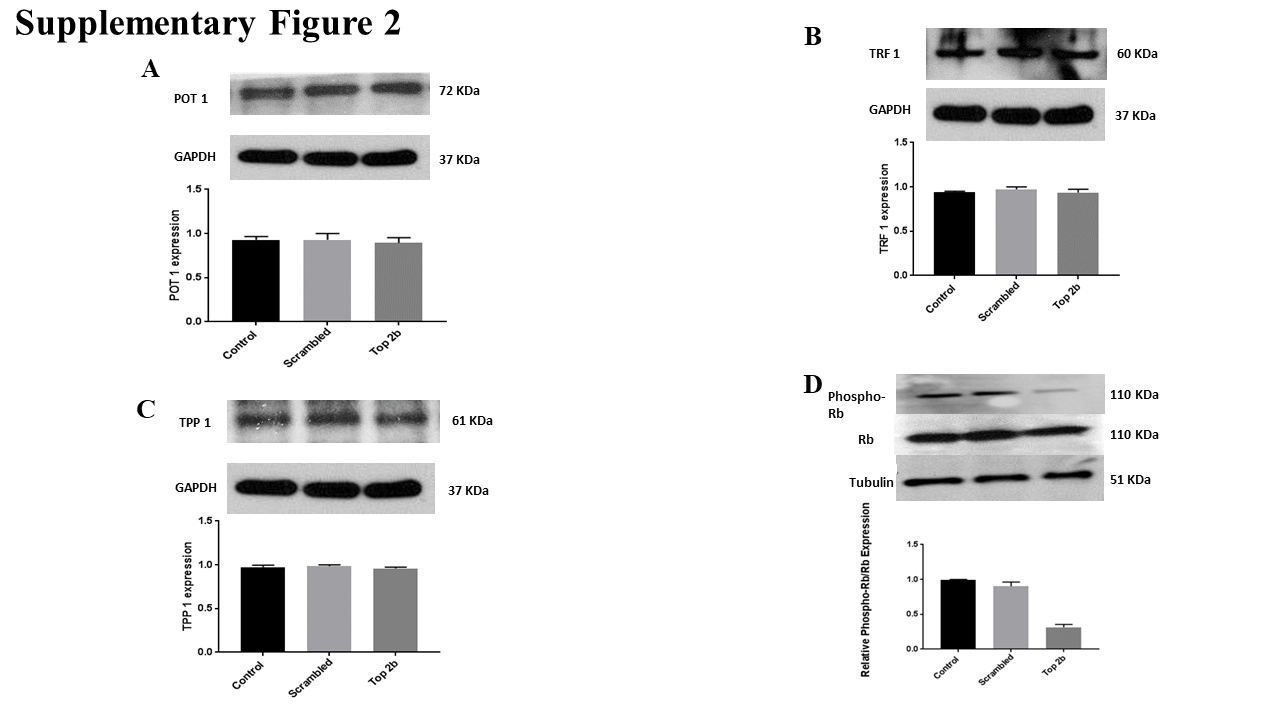

Supplement: Supplementary Figure 2 — Changes in the sheltrin complex. The other three components of shelterin complex were unchanged namely (A) POT1, (B) TRF1 as well as (C) TPP1. The end-effector of the senescence, (D) retinoblastoma protein phosphorylation was significantly decreased in Top 2b KDN H9c2 cell line indicating cells marked for senescence. [file Image_7.TIF]

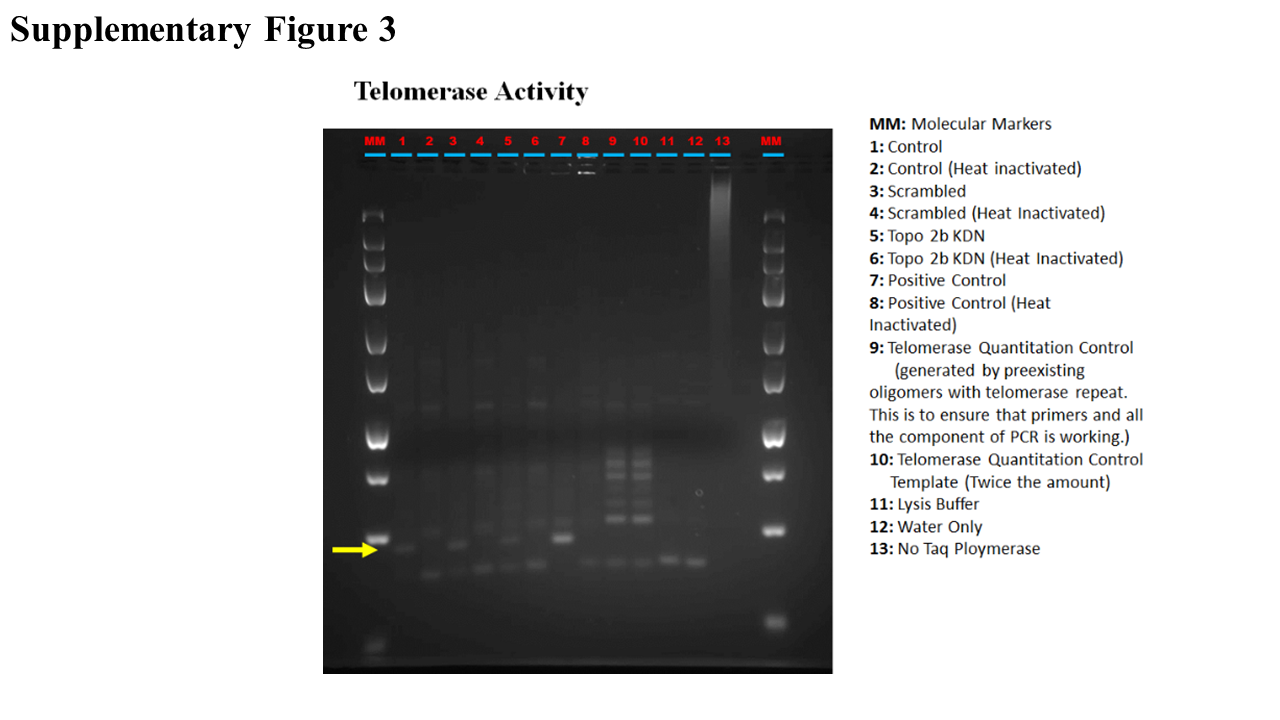

Supplement: Supplementary Figure 3 — The telomerase activity was unchanged in Top 2b KDN H9c2 cell lines when compared to control or scrambled. The activity is identified by PCR products generated by intrinsic telomerase activity. The arrow identifies the PCR product. [file Image_8.TIF]

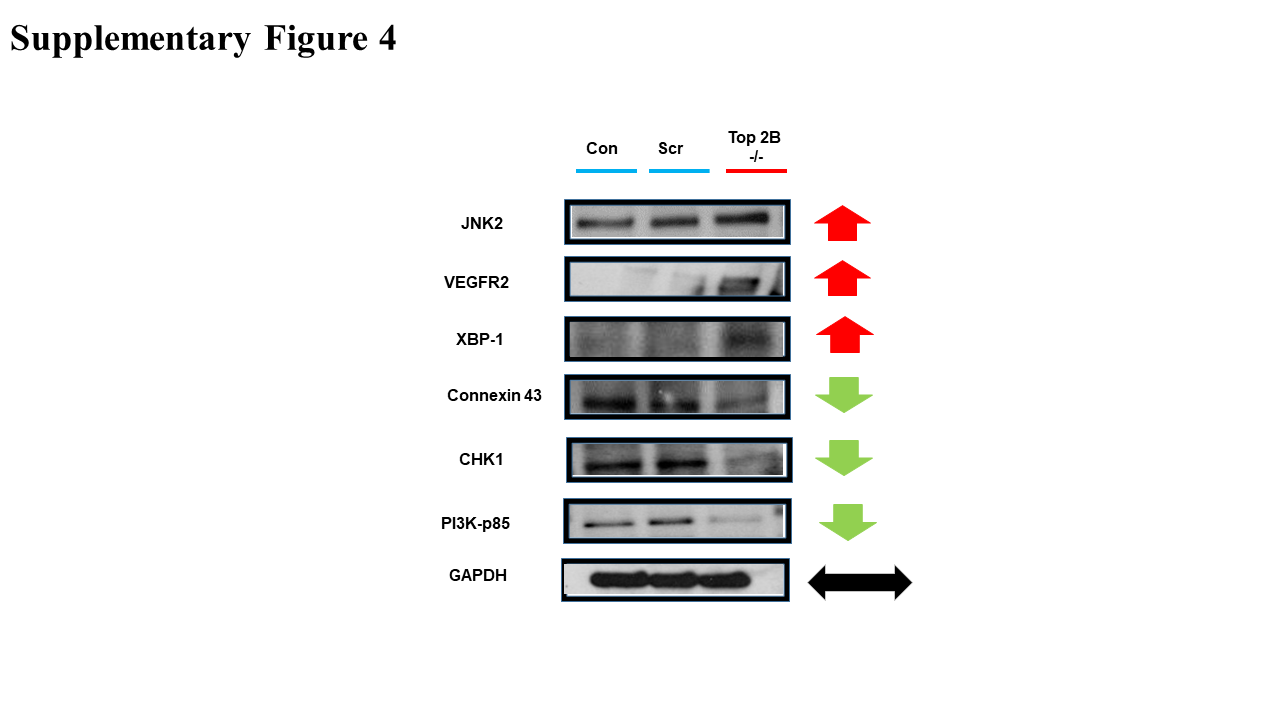

Supplement: Supplementary Figure 4 — Confirmation of changes in few protein identified in reverse phase protein analysis. There was an increase in JNK2, VEGFR2, and XBP-1 in Top2b KDN H9c2 cells when compared to scrambled or control. The connexin 43, CHK1, and PI3K were decreased in Top2b KDN H9c2 cells line when compared to its counterparts. [file Image_9.TIF]

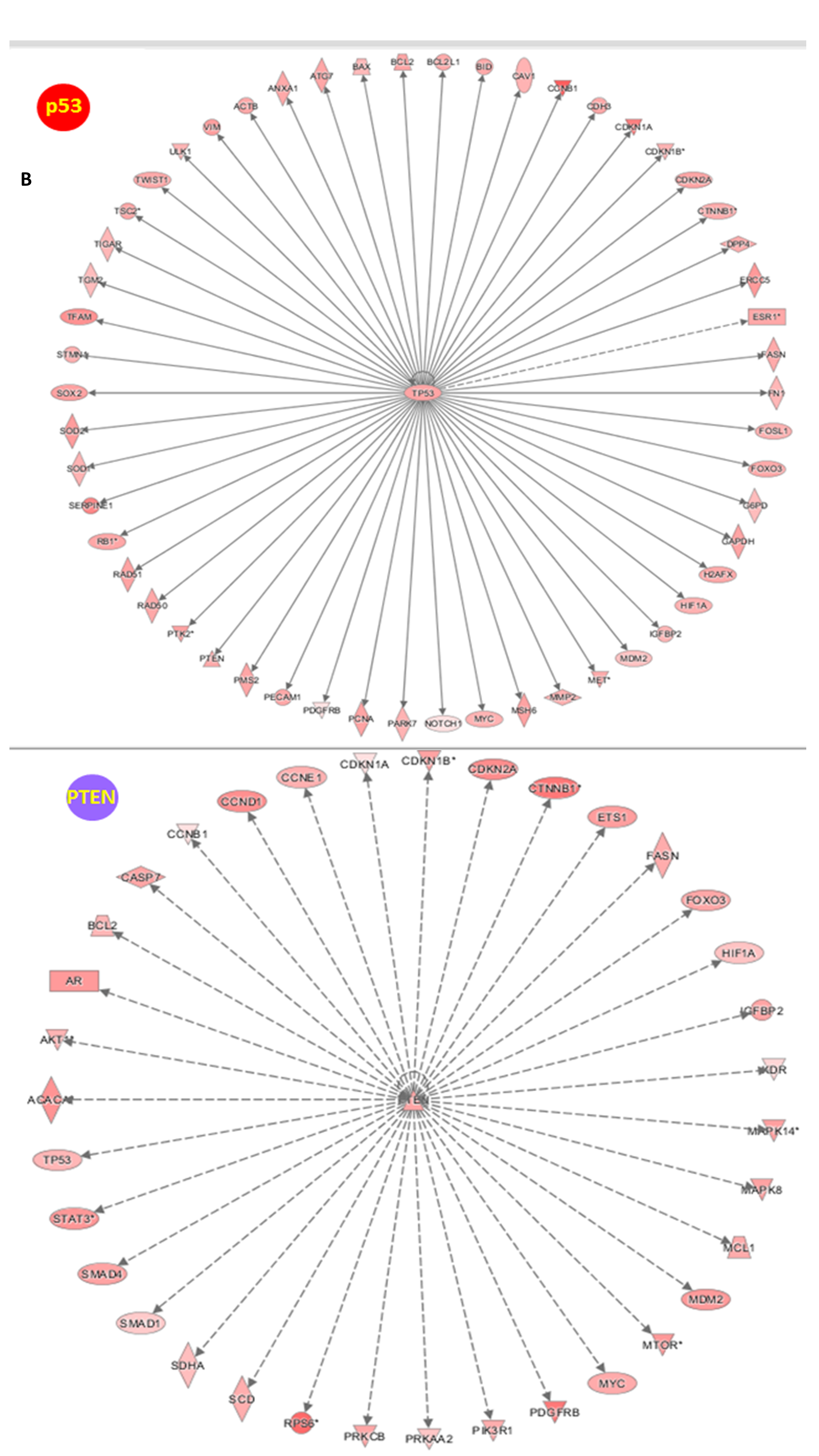

Supplement: Supplementary Figure 5 — Ingenuity Pathway analysis conducted on proteins from RPPA. The pictorial is the duplication of Figure 5B. The figure is enlarged for better viewing of the individual proteins. [file Image_10.TIF]
